# Dystonia management across Europe within ERN-RND: current state and future challenges

**DOI:** 10.1007/s00415-022-11412-4

**Published:** 2022-10-06

**Authors:** Liesanne M. Centen, David Pinter, Martje E. van Egmond, Holm Graessner, Norbert Kovacs, Anne Koy, Belen Perez-Dueñas, Carola Reinhard, Marina A. J. Tijssen, Sylvia Boesch

**Affiliations:** 1 Department of Neurology, University of Groningen, University Medical Center Groningen, PO Box 30001, 9700 RB Groningen, The Netherlands; 2 Expertise Center Movement Disorders Groningen, University of Groningen, University Medical Centre Groningen, PO Box 30001, 9700 RB Groningen, The Netherlands; 3 Department of Neurology, Medical School, University of Pécs, Rét Utca 2, Pécs, 7623 Hungary; 4 Centre for Rare Diseases and Institute of Medical Genetics and Applied Genomics, University Hospital Tübingen, Calwerstr. 7, 72076 Tübingen, Germany; 5 Department of Pediatrics, Faculty of Medicine, University Hospital Cologne, University of Cologne, Cologne, Germany; 6 Paediatric Neurology Department, Hospital Vall d’Hebron, Barcelona, Spain; 7 Department of Neurology, Center for Rare Movement Disorders Innsbruck, Medical University of Innsbruck, Innsbruck, Austria

**Keywords:** Dystonia, Europe, Survey, Dystonia management, Dystonia treatment

## Abstract

**Background:**

Since the first European-wide evaluation of dystonia management in 2016, several efforts have been made to improve dystonia-care. One of these was the development of the Dystonia Disease Group as a part of the European Reference Network for Rare Neurological Diseases (ERN-RND) that implemented several initiatives based on the recommendations made in 2016.

**Aim:**

To evaluate the current state of dystonia management across Europe.

**Methods:**

Twenty-four countries were surveyed via 62 dystonia-experts from 44 ERN-RND-related centers.

**Results:**

Dystonia-experts for adult patients were available in all surveyed countries. However, almost half of the countries evaluated accessibility as merely ‘satisfactory’. Access to genetic and neurophysiological testing was challenging to varying degrees in over half of countries. Main oral medications and botulinum toxin were available in all countries. Deep brain stimulation (DBS) was easily accessible in one-third of the countries. Dystonia research was conducted in 20/24 countries. Trainings on dystonia for general practitioners (GPs) were available in 11/24 countries. However, lack of trainings for other professionals was almost general. For pediatric dystonia, experts and specific training were available in over half of the countries.

**Conclusions:**

In this overview, we present the current state of dystonia management within ERN-RND. Management has slightly improved since 2016 in several fields, including diagnostics, availability of DBS, and research. The results highlight that future challenges in dystonia management are accessibility of experts, and diagnostic tools and treatments, education on adult and childhood dystonia, and optimization of referral pathways. These findings are important for improving dystonia care across Europe.

**Supplementary Information:**

The online version contains supplementary material available at 10.1007/s00415-022-11412-4.

## Introduction

Dystonia is a movement disorder (MD) characterized by continuous or intermittent muscle contractions leading to abnormal postures and/or movements. It is a heterogenous disorder with varying clinical features and body distribution [1]. The estimated prevalence of isolated dystonia in Europe is 16.4/100.000 [2]. Although the detected prevalence has been found to be increasing, dystonias are still labeled as rare diseases [3, 4]. Because of this, many physicians may be unfamiliar with the disease and diagnosis can be challenging [5]. This may lead to delay in diagnosis and treatment, which can intensify detrimental effects of dystonia on the health-related quality of life (HRQoL) [6–8]. Previous evaluations on the quality of dystonia management found that it did not reach the required level and therefore provided several recommendations to improve dystonia-care [9].

Recognizing the importance of improving the management of rare neurological diseases (RND), the European Union Board of Member States established the European Reference Network of Rare Neurological Diseases (ERN-RND) in 2017. This network of centers with specialist expertise in RND is designed to promote access to specialized healthcare for patients, and to facilitate collaboration on a European level between healthcare providers [10, 11]. The ERN-RND currently has 71 members, from 24 countries. Since its inception, numerous initiatives have been implemented to improve dystonia-care. Guidelines for dystonia have been developed, and the Clinical Patient Management System (CPMS), an IT platform to discuss patient cases with an expert panel, has been established. In addition, ERN-RND is closely collaborating with DystoniaNet Europe, a network with similar aims to improve dystonia-care [6].

To evaluate the current state of the management in the various ERN-RND centers, a survey was conducted among centers connected to the ERN-RND Dystonia Disease Group. The aim of this survey was to get an overview of the general access to dystonia-related healthcare providers and centers, and to gain insight into the current gaps concerning diagnostics, treatment, education, and research in the field of adult and childhood dystonia.

## Materials and methods

### The questionnaire

The survey was performed through a questionnaire designed in Google Forms and was distributed via email. It was developed by the Dystonia Disease Group of the ERN-RND, and its structure was similar to that of the questionnaire by Valadas et al. [9]. The final questionnaire consisted of 60 questions about management of dystonia and was divided into three parts:

- Part I: characterization of participants such as profession, country, main interest in dystonia, and whether the respondent took care in his/her practice of adults, children, or both.
- Part II: country characteristics including general infrastructure of dystonia healthcare, education about dystonia for care providers, ongoing dystonia research, diagnostics and treatment of dystonia, and pediatric dystonia.
- Part III: open question about the participants’ opinion on issues encountered in practice or measures suggested to improve management of dystonia in their countries.

The questionnaire can be found in Online Resource 1.

### Participants

An invitation to fill out the survey was sent to all members of the ERN-RND Dystonia Disease Group, all affiliated partners, and all applicants for full membership, a total of 60 centers (for a full list of participating centers, see Online Resource 2). Full members of ERN-RND have a certified expert-status for dystonia. Affiliated partners and applicants are MD specialists that either do currently not meet all ERN-RND preconditions or were under evaluation to become full members at the time of the invitation.

The first distribution took place in October 2020. Participants had the opportunity to complete the survey until the 10th of December 2020. Subsequently, reminders were sent out in late December 2020 and mid-January 2021 to the ERN-RND country representative if a response had not been received.

### Statistical analysis

Answers from Google Forms were exported to an Excel file. Subsequently, text information was transformed to numeric data. During this procedure, answers indicating availability of a diagnostic or therapeutic procedure was coded as 2, while unavailability as 1. For questions investigating the accessibility of experts, diagnostic and therapeutic procedures (e.g., difficult, satisfactory or easy) or intervals between main milestones of establishing the diagnosis of dystonia such as the interval between the first evaluation and molecular diagnosis, Likert type scales were used to scale responses (e.g., 1 = difficult, 2 = satisfactory, 3 = easy or 1 = less than 1 year, 2 = 1–2 years, 3 = 3–4 years, 4 = more than 4 years, etc.). Finally, numeric data were descriptively analyzed in Microsoft Office Excel 2016 (Microsoft Cooperation, Redmond, WA, USA). Data were analyzed both on country level and by combining data of all respondents irrespective of country.

## Results

### Characteristics of respondents

Finally, sixty-two respondents from 44 centers (73%) linked to ERN-RND located in 24 different European countries participated in the survey. All countries represented in ERN-RND participated. All respondents were physicians with expertise in the field of MDs. The majority of them were neurologists (*n* = 51), followed by child neurologists (*n* = 10), and one neurosurgeon. More than half (51.6%, *n* = 32) of the respondents reported to provide care for children in their practice. The main areas of interest in dystonia of the participants were clinical (41.9%, *n* = 26), DBS (22.6%, *n* = 14), botulinum toxin (17.7%, *n* = 11), genetics (9.7%, *n* = 6) and imaging (3.2%, *n* = 2), respectively.

**Fig. 1:**
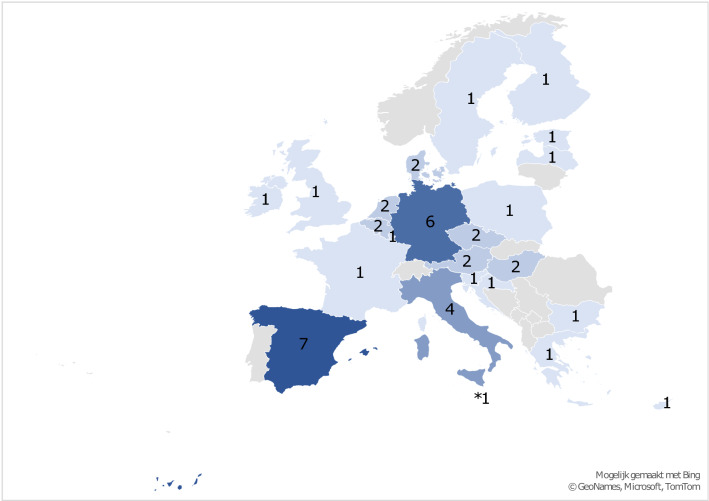
Number of participating centers per country in the ERN-RND survey. Participating countries, with the number of respondents in brackets: Bulgaria (*n* = 1), Czech Republic (*n* = 3), France (*n* = 3), Germany (*n* = 8), Hungary (*n* = 3), Italy (*n* = 6), Netherlands (*n* = 3), Poland (*n* = 3), Slovenia (*n* = 1), Spain (*n* = 9), United Kingdom (*n* = 1), Austria (*n* = 4), Croatia (*n* = 1), Denmark (*n* = 3), Estonia (*n* = 1), Finland (*n* = 2), Latvia (*n* = 1), Luxembourg (*n* = 1), Malta (*n* = 1), Belgium (*n* = 2), Cyprus (*n* = 1), Greece (*n* = 1), Ireland (*n* = 2), Sweden (*n* = 1)

The number of respondents of ERN-RND dystonia teams per country varied from 1 to 9. Highly represented countries included Spain (*n* = 9), Germany (*n* = 8), Italy (*n* = 6) and Austria (*n* = 4). Respondents from these countries comprised 43.5% of the total participant population (Fig. 1).

### Availability and accessibility of dystonia experts

MD experts for adult patients were reported to be available in all included countries.

Reported accessibility of dystonia experts was easy or satisfactory in the majority of cases (8.3% and 45.8% respectively), while a minority of countries reported that dystonia experts were with difficulty available (16.7%). In some countries, accessibility of experts varied among regions (Table 1).

**Table 1:** Accessibility of dystonia experts per country

Country	No. centers	Accessibility of dystonia experts*			Overall evaluation of accessibility of dystonia experts
		Difficult	Satisfactory	Easy	
Austria	2	0	4	0	Satisfactory
Belgium	2	0	1	1	Brussels: satisfactory, Leuven: easy
Bulgaria	1	0	1	0	Satisfactory
Croatia	1	0	1	0	Satisfactory
Cyprus	1	0	1	0	Satisfactory
Czech Republic	2	2	1	0	Prague I: difficult/satisfactory, Prague II: difficult
Denmark	2	2	1	0	Aarhus: difficult, Copenhagen: difficult/satisfactory
Estonia	1	0	0	1	Easy
Finland	1	0	0	2	Easy
France	2	1	2	0	Paris: satisfactory, Lille: difficult
Germany	6	2	5	1	Aachen: satisfactory, Hannover: difficult, Lübeck: satisfactory, München: difficult, Tübingen: satisfactory/easy, Würzburg: satisfactory
Greece	1	0	1	0	Satisfactory
Hungary	2	3	0	0	Difficult
Ireland	1	0	2	0	Satisfactory
Italy	4	1	5	0	Bologna: difficult/satisfactory, Padova: satisfactory, Rome: satisfactory, Siena: satisfactory
Latvia	1	0	1	0	Satisfactory
Luxembourg	1	1	0	0	Difficult
Malta	1	0	1	0	Satisfactory
Netherlands	2	0	3	0	Satisfactory
Poland	1	3	0	0	Difficult
Slovenia	1	1	0	0	Difficult
Spain	7	1	7	1	Barcelona: easy (1), satisfactory (3), Madrid I: satisfactory, Madrid II: satisfactory, Oviedo: satisfactory, Santander: satisfactory, Vall d’Hebron: satisfactory
Sweden	1	0	1	0	Satisfactory
United Kingdom	1	0	1	0	Satisfactory

Values indicate the number of respondents who rated accessibility of dystonia experts as difficult, satisfactory, and easy

### Availability of tertiary centers

Tertiary centers for dystonia exist in all but three (Greece, Luxembourg, and Slovenia) countries. Remarkably, tertiary care centers specifically for DBS in dystonia were present in 22 (91.7%) countries, only Latvia and Luxembourg were exceptions. Respondents from 10 countries (37.5%; Austria, Cyprus, Denmark, Estonia, Finland, Greece, Latvia, Malta, Slovenia, and the United Kingdom) indicated that patients with dystonia had at least one visit in a tertiary center. In addition, in seven countries (29.2%; Czech Republic, France, Germany, Ireland, Italy, the Netherlands, Spain), some but not all patients were seen at least once in tertiary centers. Finally, respondents from seven countries (33.3%; Belgium, Bulgaria, Croatia, Hungary, Luxembourg, Poland, Sweden) reported that patients did generally not visit tertiary centers at least once, despite being available in the majority of these countries.

### Diagnostics for dystonia

All ancillary testing methods that were assessed in the survey (genetic testing, neurophysiological tests such as electromyography and transcranial magnetic stimulation, and magnetic resonance imaging—MRI) were available to some degree in all surveyed countries. Genetic testing was generally easily available in ten countries (Belgium, Croatia, Denmark, Estonia, Finland, Ireland, Luxembourg, the Netherlands, Slovenia, and Sweden). Additionally, in seven countries this was easily available in some regions, but with some difficulty in other regions (Austria, Czech Republic, France, Germany, Hungary, Italy, and Spain). In the remaining countries, one reason for difficult access was an issue with reimbursement of testing costs. Easy availability of neurophysiological testing was reported by 11 countries (Bulgaria, Croatia, Cyprus, Denmark, Estonia, Finland, Germany, Hungary, Luxembourg, the Netherlands, and Sweden). MRI was easily available in all countries except for Italy, where the majority reported easy access except for one participant.

**Fig. 2:**
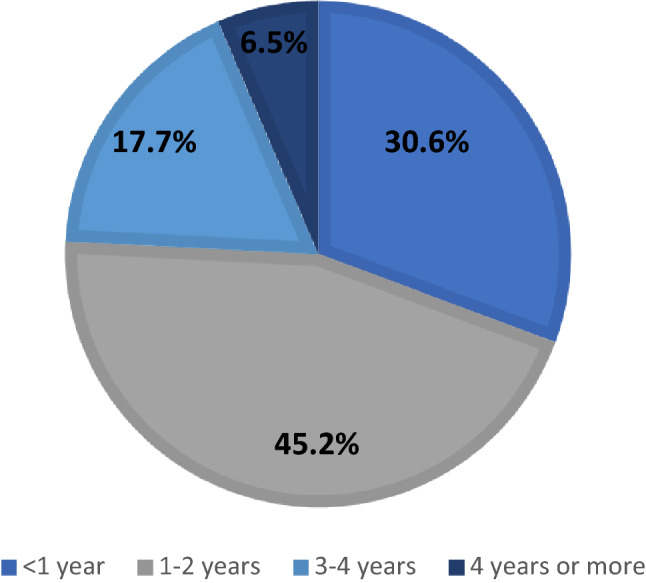
Mean time between the first appearance of symptoms of dystonia to evaluation by a movement disorders expert (by % of respondents)

Almost one-third (30.6%) of respondents reported that dystonia patients are evaluated by an MD-expert within a year after the onset of symptoms (see Fig. 2). Seventeen respondents (27.8%) indicated that it usually takes less than 1 year from first evaluation to establishing a molecular diagnosis, followed by 3–4 years by 15/61 (24.6%), 1–2 years by 14/61 (22.9%), and 4 years or longer by 11/61 (18.0%) respondents.

### Non-surgical treatment modalities

One or more of the main oral medications for dystonia including anticholinergics (e.g., biperiden, trihexyphenidyl), muscle relaxants (e.g., baclofen), benzodiazepines (e.g., clonazepam), antipsychotics (e.g., tiapride) and vesicular monoamine transporter inhibitors (e.g., tetrabenazine) were easily available in all participating countries. Botulinum toxin was also easily available in the majority of countries, however, a minority of participants from several countries (Croatia, Cyprus, Czech Republic, Denmark, France, Germany, Greece, Italy, Luxembourg, Poland, and Spain) reported some difficulty with access in some regions. Other types of therapies such as physical therapy and rehabilitation, speech therapy, and occupational therapy and allied healthcare professionals including social care workers, psychologists, geneticists, and psychiatrists were available in almost all surveyed countries to varying degrees (for additional data see Online Resource 3).

Data on the availability of different types of technical devices for dystonia patients in surveyed countries can be found in Online Resource 4.

### Surgical treatment modalities

DBS as treatment for dystonia was available in all but one country (Latvia). In Ireland DBS was available, but not in all country regions. In countries where DBS was generally available, its accessibility varied. It was easily accessible in seven (29.2%) and with some difficulty in eight (33.3%) countries, while in the other countries, accessibility of DBS varied among country regions (Table 2). DBS expert neurologists and DBS neurosurgeons were reported to be unavailable only in Latvia.

**Table 2:** Accessibility of deep brain stimulation per country

Country	Accessibility of deep brain stimulation*			Overall evaluation of accessibility of deep brain stimulation
	Easy	With some difficulty	Not available	
Austria	3	1	0	In some regions easy, in some with difficulty
Belgium	1	0	0	Easy
Bulgaria	0	1	0	With some difficulty
Croatia	0	1	0	With some difficulty
Cyprus	0	1	0	With some difficulty
Czech Republic	1	2	0	In some regions easy, in some with difficulty
Denmark	0	3	0	With some difficulty
Estonia	1	0	0	Easy
Finland	1	1	0	In some regions easy, in some with difficulty
France	2	1	0	In some regions easy, in some with difficulty
Germany	5	3	0	In some regions easy, in some with difficulty
Greece	0	1	0	With some difficulty
Hungary	3	0	0	Easy
Ireland	0	1	1	In some regions with difficulty, in some not available
Italy	2	4	0	In some regions easy, in some with difficulty
Latvia	0	0	1	Not available
Luxembourg	1	0	0	Easy
Malta	1	0	0	Easy
Netherlands	3	0	0	Easy
Poland	0	3	0	With some difficulty
Slovenia	0	1	0	With some difficulty
Spain	5	4	0	In some regions easy, in some with difficulty
Sweden	0	1	0	With some difficulty
United Kingdom	1	0	0	Easy

Values indicate the numbers of respondents who rated accessibility of deep brain stimulation as easy, difficult or not available

The most frequently reported cause of dystonia in patients who underwent DBS was isolated dystonia (50/58, 86.2%), followed by acquired dystonia (4/58, 6.9%). Almost two-thirds of the participants (38/61, 62.3%) indicated that less than 5% of dystonia patients at their centers undergo DBS. Most of these patients were over 20 years of age at the time of DBS surgery (Table 3).

**Table 3:** Mean age of patients at time of deep brain stimulation surgery (based on answers of 55/62 respondents)

Country	Mean age at DBS implantation			
	6–10 years	10–15 years	15–20 years	> 20 years
Austria	0	2	0	2
Belgium	0	0	0	2
Bulgaria	0	0	0	1
Croatia	0	0	0	1
Cyprus	0	0	0	1
Czech Republic	0	1	0	2
Denmark	0	1	0	2
Estonia	0	0	0	1
Finland	2	0	0	0
France	0	0	1	2
Germany	1	2	0	5
Greece	0	0	0	1
Hungary	0	0	0	3
Ireland	0	0	0	1
Italy	0	3	1	1
Latvia	0	0	0	0
Luxembourg	0	0	0	0
Malta	0	0	0	1
Netherlands	0	2	0	1
Poland	0	0	1	2
Slovenia	0	1	0	0
Spain	1	1	0	5
Sweden	0	0	0	0
UK	0	0	0	1
Total	4	13	3	35

Stereotactic lesioning was easily available only in three countries (Belgium, Finland, and Malta), in the majority of countries with difficulty, and was reported to be unavailable in six countries (Croatia, Cyprus, Denmark, Estonia, Greece, and Latvia).

### Education for dystonia healthcare providers

Regarding education on MDs in general, internships for neurology residents were available in all but seven countries (Estonia, Greece, Latvia, Malta, Poland, Slovenia, and Sweden). Also, the majority of participants (54/61, 88.5%) from 22 countries reported available teaching courses or symposia for residents and general neurologists on MDs, while less than half of the respondents (29/60, 48.3%) from 15 countries indicated availability of these to general practitioners (GPs). Teaching courses on dystonia for GPs or pediatricians were available in 11 countries (45.8%), while courses on DBS for dystonia were only available in some regions of seven countries (33.3%).

Education for other healthcare providers involved in dystonia-care was also assessed. Reported availability of specific teaching courses on MDs were 19/60 (31.6%) from 13 countries for nurse practitioners, 17/60 (28.3%) from 11 countries for speech therapists, and 24/59 (40.7%) from 13 countries for physiotherapists, respectively. For specific results on teaching courses for dystonia and DBS, see Table 4.

**Table 4:** Availability of specific educational training on dystonia and deep brain stimulation for healthcare providers

Country	Specific educational training on									
	Dystonia					Deep brain stimulation				
	Residents and general neurologists	Clinical nurse specialists	Physiotherapists	Speech therapists	General practitioners and pediatricians	Residents and general neurologists	Clinical nurse specialists	Physiotherapists	Speech therapists	General practitioners and pediatricians
Austria	+	+ *			+	+ *				+ *
Belgium	+		+ *	+ *			+ *			+ *
Bulgaria	+				+	+				
Croatia	+				+	+	+			
Cyprus	+		+							
Czech Republic	+ *	+ *	+ *			+ *	+ *	+ *	+ *	
Denmark	+ *	+ *	+	+ *	+ *	+ *	+		+ *	+ *
Estonia	+	+	+			+				
Finland	+	+ *	+ *	+ *	+ *	+	+ *			
France	+	+ *	+ *	+ *	+ *	+	+ *			
Germany	+ *	+ *	+ *	+ *	+ *	+ *	+ *	+ *	+ *	+ *
Greece	+					+				
Hungary	+	+ *	+ *			+	+ *			+ *
Ireland	+	+ *					+ *	+ *	+ *	
Italy	+	+ *	+ *	+ *	+ *	+ *	+ *	+ *	+ *	+ *
Latvia										
Luxembourg	+					+				
Malta								+		
Netherlands	+	+ *	+	+ *	+ *		+			
Poland	+ *					+ *				
Slovenia	+									
Spain	+	+ *	+ *	+ *	+ *	+ *	+ *	+ *	+ *	+ *
Sweden	+			+		+				
United Kingdom	+	+	+	+	+	+	+	+		

A + sign indicates easy availability. Combined with an asterisk it indicates that it was not available in all regions of the country

### Dystonia related research

In 20 (83.3%) out of 24 countries, there was at least 1 type of dystonia research currently ongoing. Clinical research on dystonia was performed in 18/24 countries (75%), and clinical research specifically aimed at DBS for dystonia was carried out in 14/24 countries (58.3%). The numbers of countries that were conducting research on genetics, basic research, imaging, and neuropsychology were 15 (62.5%), 11 (45.8%), 11 (45.8%), and 8 (33.3%), respectively.

Other types of ongoing research that were mentioned included research for physiotherapy in dystonia (the Netherlands) and transcranial magnetic stimulation (Spain). Latvia, Luxembourg, Malta, and Sweden reported that there was currently no type of dystonia research performed (Table 5).

**Table 5:** Different types of research on dystonia per country

Country	Research type						
	Basic	Clinical	Genetics	DBS for dystonia	Neurophysiology	Imaging	Other
Austria	+	+	+	+		+	
Belgium	+	+	+	+			
Bulgaria	+	+					
Croatia			+				
Cyprus		+					
Czech Republic		+	+	+	+	+	
Denmark		+	+	+		+	
Estonia		+					
Finland	+	+	+	+			
France	+	+	+	+	+	+	
Germany	+	+	+	+	+	+	
Greece				+			
Hungary	+	+	+	+		+	
Ireland		+	+		+	+	
Italy	+	+	+	+	+	+	
Latvia							
Luxembourg							
Malta							
Netherlands	+	+	+	+	+	+	+ (Physiotherapy)
Poland		+					
Slovenia		+	+	+			
Spain	+	+	+	+	+	+	+ (rTMS)
Sweden							
United Kingdom	+	+	+	+	+	+	
Total number of countries with ongoing research on a field	11	18	15	14	8	11	2

A ‘ + ’ sign indicates that this type of research was carried out in the respective country

### Diagnostic and treatment guidelines for dystonias, MD societies and patient associations

National diagnostic and therapeutic guidelines for dystonia were available in half (*n* = 12) of the included countries. However, in six (Austria, Czech Republic, Denmark, Germany, Italy, and Poland) countries not all respondents were aware of the existence of these guidelines. Specific guidelines on DBS for dystonia were reported to be available in 10 (43.4%) of 23 countries. The existence of such guidelines was also unknown to some respondents in seven countries (Austria, Czech Republic, Denmark, Germany, Hungary, Italy, and Poland).

In all but five countries (Croatia, Cyprus, Latvia, Malta, Slovenia), an MD society or working group existed. Network groups of experts for dystonia and specifically for DBS in dystonia were available in 19 (unavailable in Estonia, Greece, Luxembourg, Malta, and the United Kingdom) and 15 (unavailable in Bulgaria, Croatia, Estonia, Greece, Latvia, Luxembourg, Malta, Slovenia, and the United Kingdom) countries, respectively. A national patient association for dystonia was present in 15 countries (unavailable in Bulgaria, Croatia, Cyprus, Estonia, Greece, Latvia, Luxembourg, Malta, and Slovenia).

For a comparison of several aspects of dystonia care from the 2016 survey to the current survey see Table 6.

**Table 6:** Comparison of availability of several aspects of the 2016 survey results and the current survey results

	2016 survey	Current survey
Expertise		
MD experts	23/24 (96%)	24/24 (100%)
Tertiary care centers	21/24 (87.5%)	21/24 (87.5%)
Training		
MD teaching courses/symposia for neurologists and residents	24/24 (100%)	22/24 (91.7%)
MD internships for neurology residents	10/24 (41.7%)	17/24 (70.8%)
MD teaching courses for GPs	12/24 (50%)	15/24 (62.5%)
Specific training on MDs	Nurses: 10/24 (41.7%) Physiotherapists: 12/24 (50%) Speech therapists: 7/24 (29.2%)	Nurse practitioners: 13/24 (54.2%) Physiotherapists: 13/24 (54.2%) Speech therapists: 11/24 (45.8%)
Ancillary tests		
Genetic testing	Easy: 12/24 (50%) Difficult: 9/24 (37.5%) Not available: 3/24 (12.5%)	Easy: 10/24 (41.7%) Varying availability between regions (easy or difficult): 7/24 (29.2%) Difficult: 7/24 (29.2%)
Electrophysiological testing	Easy: 11/24 (45.8%) Difficult: 11/24 (45.8%) Not available: 2/24 (8.3%)	Easy: 11/24 (45.8%) Varying availability between regions (easy or difficult): 8/24 (33.3%) Difficult: 5/24 (20.8%)
Treatment		
Botulinum toxin	Easy: 18/24 (75%) Difficult: 6/24 (25%)	Easy: 12/23 (52.2%) Difficult: 5/23 (21.7%) Varying availability between regions (easy or difficult): 6/23 (26%)
Deep brain stimulation	Easy: 13/24 (54.2%) Difficult: 5/24 (20.8%) Not available: 6/24 (25%)	Easy: 7/24 (29.2%) Difficult: 8/24 (33.3%) Varying availability within country: -Easy or difficult: 7/24 (29.2%) - Difficult or not available 1/24 (4.2%) - Not available 1/24 (4.2%)
Research		
Clinical trials on dystonia	19/24 (79.1%)	18/24 (75%)
At least 1 type of dystonia research	21/24 (87.5%)	20/24 (83%)
Societies and associations		
Local MD society	19/24 (79.2%)	19/24 (79.2%)
Dystonia networking group	13/24 (54.2%)	19/24 (79.2%)
Dystonia patients’ association	16/24 (66.7%)	15/24 (62.5%)

### Dystonia in children

Based on data from 21 countries, MD experts were available for children with dystonia with the exception of Latvia and Poland. DBS expert child neurologists were unavailable in Cyprus, Latvia, Poland, and Slovenia. About a third (15/48, 31.3%) of respondents had access to an official MD working group for children in six countries (Finland, France, Germany, Italy, the Netherlands, Spain).

Regarding education, teaching courses on pediatric MDs for residents and general neurologists were available to 37/53 (69.8%) respondents from 14 out of 21 countries (Belgium, Bulgaria, Czech Republic, Denmark, Estonia, Finland, France, Germany, Hungary, Ireland, Italy, the Netherlands, and Spain). Additionally, specific teaching courses or symposia about pediatric dystonia were available to 31/58 (53.4%) participants in 12 out of 22 countries (Austria, Belgium, Denmark, Finland, France, Germany, Hungary, Ireland, Italy, Luxembourg, Spain, and the United Kingdom). For GPs, courses on pediatric MDs were available in a minority of cases (12/50, 24.0%) in Belgium, Czech Republic, Estonia, Finland, Germany, Hungary, Italy, and Spain. Internships on MDs in children for residents in pediatrics were available to 19/54 (35.2%) of respondents from Austria, Bulgaria, Croatia, Denmark, France, Germany, Hungary, Ireland, Italy, the Netherlands, and Spain.

Only about one-third (36.4%) of surveyed dystonia teams initiate DBS treatment usually in childhood. In Finland and Slovenia, all respondents indicated that dystonia patients undergo DBS implantation mainly in childhood. DBS implantation was reported to be performed rather in childhood than in adulthood in Italy and the Netherlands (Table 3).

### Additional opinions of respondents

The most frequently reported measures to be implemented concerned improving availability of genetic testing (Austria, Bulgaria, Czech Republic, Hungary, Finland, France, Germany, Greece, Italy, Latvia, Malta, Poland, Spain, the United Kingdom); implementation of multidisciplinary teams/programs (Belgium, Bulgaria, Croatia, Cyprus, Denmark, Finland, Germany, Italy, Latvia, Luxembourg, the Netherlands, Poland, Spain); more widespread availability of botulinum toxin clinics (Czech Republic, France, Germany, Hungary, Ireland, Italy); national and European (pediatric) patient registries (Austria, Belgium, Croatia, Czech Republic, Finland, Germany, Greece, Italy, Luxembourg, Malta, the Netherlands, Spain); guidelines on diagnosis and treatment of dystonia (Finland, Spain); and measures to increase awareness of dystonia in primary care (Czech Republic, Germany, Greece, Italy, Latvia, Spain, and the United Kingdom).

## Discussion

The results of this survey offer an updated overview of the state of access to diagnostics, treatment, research, and education in the field of dystonia in various centers linked to the ERN-RND Dystonia Group.

Globally, the accessibility of dystonia experts was evaluated as merely ‘satisfactory’ in our study, which was similar to previous surveys [9, 11]. Possible reasons could be the lack of available experts in certain country regions, experts only being available in tertiary centers, and inadequate national referral systems [9]. An interesting observation was that in large countries with multiple ERN-RND centers (France, Germany, Italy, Spain) a majority reported satisfactory access, while a small minority reported difficult access. Surprisingly, even experts from the same center gave contradictory answers about their experience with access to dystonia-experts. This could indicate that knowledge regarding referral pathways or location of centers even within an expert group is not optimal. Or this could be due to the general phrasing of the question in the survey, allowing for a broad interpretation. This is supported by the comments, in which a spectrum of reasons were mentioned to support their answers among which the number and distribution of experts, center locations, and referral pathways.

Regarding availability of tertiary centers, our survey shows a higher number of countries (7/24 vs. 10/24) where patients visit tertiary centers at least once during the course of their dystonia than previously reported [9]. This can be considered an important step towards improving the quality of dystonia-care, because some diagnostic tools and treatment options that are highly effective in achieving clinically important improvements in the severity of dystonia, experienced disability, and the HRQoL such as DBS, are usually only available in such centers [12].

Every participating country had access to genetic and electrophysiological testing. However, access to both testing methods was a challenging issue in several regions of more than half of the surveyed countries. This was in line with the 2016 survey [9]. However, previously some of these testing methods were unavailable in two European countries, which was no longer the case in our study. Nonetheless, further efforts are required to improve wider applicability of genetic and neurophysiological testing. These efforts should be especially aimed at overcoming financial and reimbursement issues and improving availability of professionals trained to use these diagnostic tools [11]. Although a diagnosis of dystonia is primarily based on expert clinical evaluation, these diagnostic measures can aid in further specifying the disorder, allowing treatment to be more tailor-made to the needs of the patient [13].

Regarding treatment options for dystonia, oral medications were easily available in all participating countries. Botulinum toxin injections were also available everywhere. However, a minority of participants from various countries reported to have some difficulty with access to botulinum toxin in some regions. A possible explanation for this could be the disproportionate number of trained physicians available to administer botulinum toxin. More widespread availability of botulinum toxin clinics was mentioned as an improvement point for dystonia care by several participants. Overall, the accessibility of oral medications did not change, whilst we detected a somewhat worse accessibility of botulinum toxin [9]. However, it cannot be excluded that the latter results from the partly different groups of surveyed countries and centers.

Considering surgical treatment options for dystonia, DBS was found more widely available in ERN-RND than in the 2016 survey. Easy accessibility of DBS was found to be less frequently reported compared to a previous survey [11]. Cross-border referral, which is available via ERN-RND could be a first step of resolving the issue in countries or regions where DBS is currently unavailable [9]. Compared to DBS, lesioning surgery was generally less available. This might reflect the fact that DBS is nowadays the mainstay of surgical treatment for dystonia which, in turn, might have led to a reduced interest and expertise in lesioning procedures.

Training, internships, and educational courses are important aspects in improving recognition of dystonia in both primary care and by general neurologists. Teaching courses for general neurologists and residents in neurology were widespread available. However, teaching courses on MDs for GPs were available according to merely 40% of respondents from less than two-thirds of participating countries. We found a slight improvement in the number of countries providing such trainings to GPs compared to the previous surveys [9, 11]. However, considering that GPs play the role of gatekeepers in most European health care systems regarding referral to specialist care, the lack of appropriate education on MDs can be considered an important issue. This might lead to delayed recognition of the disease and refrain patients from timely diagnosis and treatment.

Concerning dystonia research, clinical research was performed most often, followed by genetics, imaging, and neurophysiology which is similar to the 2011 survey [11]. The Netherlands, France, Italy, and Denmark kept their leading roles in scientific investigation of dystonia by conducting the most types of research. We also detected novel dystonia research activity in some countries (e.g., in Bulgaria). International networks such as ERN-RND might be notable contributors to this beneficial change by helping the initiation of collaborative studies crossing the borders of European countries. We detected no significant change considering the number of countries engaging in at least one area of dystonia research or in the distribution of different types of dystonia research [9].

Our survey also tried to capture the quality of dystonia management in children. Comparison of results at this point cannot be made, because such data were not collected in the survey by Valadas et al. However, our data show that availability of dystonia experts for children and educational opportunities on child dystonias are currently more limited than in adult care in several European countries such as Czech Republic, Hungary, Ireland and Poland. Similarly, with the exception of Finland, Italy, the Netherlands and Slovenia, DBS for dystonia is also more widely used in adult patients than in children.

When interpreting results of this survey, possible differences in the healthcare systems of included countries should be considered. Longer waiting times, fewer available healthcare providers per population and shortages or uneven geographical distribution of specialists may partly contribute to difficult accessibility of dystonia experts in some countries (e.g., Hungary, Poland, and Slovenia) [14]. Differences in available public sources and annual quota for some treatments (e.g., botulinum toxin, DBS) among surveyed countries may also partly explain varying degrees of difficulty in dystonia care across Europe [14]. In addition, higher out-of-pocket health expenditure may limit some fields of dystonia care to a greater extent in various countries (e.g., Bulgaria, Greece, Latvia, Malta) compared to those with broader public funding of healthcare (e.g., Austria, Belgium, Croatia, Czech Republic, Denmark, Hungary) [15]. Although ERN-RND countries show promising improvements in the field of dystonia management, to sustain the gained results and to achieve further improvement, the integration of ERN-RND into different national healthcare systems of the European countries in a systematic, sustainable, and financed manner would be essential. This remains an overarching effort by the ERN-RND.

The strength of the present survey lies in its evaluation on a European-wide level by including experts who are playing an important role in the local management of dystonias. However, several limitations should be taken into account regarding this study. First, the generalizability of our results might be affected by varying numbers of responses from different countries. This leads to the fact that a minority of participating countries makes up approximately half of all respondents. The reason behind this is that in some countries only one ERN-RND center has been assigned by national authorities, while other EU countries harbor multiple ERN-RND linked centers. A challenge lies ahead in the future to improve generalizability of results for countries with only one ERN-RND center. Potential ERN-RND centers undergo a thorough national and EU wide evaluation process before they can be assigned as such. Consequently, in some countries no other potential centers are present or do not meet the qualifications to become an ERN-RND center. Possibly, in the future efforts could be undertaken to have more physicians participate in the survey from the same center.

Another issue might be that for several questions, various respondents from the same country reported different answers. Therefore, conclusions may be less nuanced from countries with only one center which might mean among others that possible inter-regional differences in some countries could have remained uncovered. Different answers from the same country might result from either these aspects of dystonia care not being available to all healthcare providers, or not being accessible in all regions of that country. Additionally, it should be taken into account that in some countries children with dystonia are treated by pediatricians and might not be referred to specialized centers until they reach adulthood. Therefore, the experience from physicians treating children might differ significantly from those treating adults.

However, the inclusion of more centers and more experts from a single country can also be regarded as a strength of our survey because it could detect differences between country regions. In addition, the participants in this survey were members or aspiring members of ERN-RND at the time when the survey was conducted and might therefore have better access in general to dystonia healthcare compared to peripheral hospitals in the respective country. Consequently, the data could be biased towards a more positive outlook on access to dystonia-care. Nevertheless, the ERN-RND network consists of experts in the field of dystonia. It can be reasonably expected for respondents to be well aware of the management of dystonia in their countries.

Finally, since this survey was sent out during the height of the COVID-19 pandemic, we expect that efforts initiated since the 2016 survey to improve dystonia care will undoubtedly have been delayed, disrupted, or altered. As the pandemic severely impacted regular dystonia care, this probably also applied to efforts to improve it [16]. Therefore, we might argue that the reassessment of dystonia care after 4 years is quite soon to see substantial results. Moreover, the pandemic also led to a paradigm shift in (digital) communication. It paved the way for developing telemedicine systems in many hospitals, which not only provided a new medium for consulting doctors but also facilitates contact between experts on a national or international level. Telemedicine as a subject was not included in this survey and should be considered in future endeavors to assess dystonia care.

Based on the challenges we have identified through this survey we established several key priorities to further improve dystonia care in Europe.

1. Optimization of information supply to healthcare providers about referral pathways for dystonia patients within each country on all healthcare levels ranging from primary to tertiary care.
2. Further improving the accessibility of genetic and neurophysiological testing to aid in diagnosis of dystonia.
3. Working towards widespread availability of healthcare providers trained in administration of botulinum toxin treatment.
4. Improvement of accessibility to DBS, in the short term by optimization of referral pathways within countries and by raising attention to the existence of cross-border referral systems such as provided through the ERN-RND, and in the long-term by expanding the number of specialists providing DBS.

## Conclusions

To conclude, several aspects of dystonia management show promising improvements since the inception of ERN-RND, whilst some weaknesses are still addressed insufficiently. On one hand, this may indicate that efforts made by international networks such as ERN-RND are justified. On the other hand, some future missions still lie ahead of us with regard to improving access to diagnostic tools, treatment modalities, and training opportunities.

## Supplementary Information

Below is the link to the electronic supplementary material.

Supplementary file1 (PDF 615 KB)

Supplementary file2 (DOCX 22 KB)

Supplementary file3 (DOCX 23 KB)

Supplementary file4 (DOCX 21 KB)

## Abbreviations

COST: European Cooperation in Science and Technology
DBS: Deep brain stimulation
ERN-RND: European Reference Network for Rare Neurological Diseases
GPs: General practitioners
HRQoL: Health-related quality of life
MD: Movement disorders
MRI: Magnetic resonance imaging
