# Supplementary material for: Dystonia management across Europe within ERN-RND: current state and future challenges
Source: J Neurol. 2022 Oct 6;270(2):797–809. doi: 10.1007/s00415-022-11412-4 (PMC9540051; doi:10.1007/s00415-022-11412-4)
Supplement: Supplementary file 1 — Supplementary file1 (PDF 615 KB) [file 415_2022_11412_MOESM1_ESM.pdf]

Dystonia management across Europe within ERN-RND: current state and future challenges

Journal of Neurology

Liesanne M. Centen, MD*; David Pinter, MD, PhD*; Martje E. van Egmond, MD, PhD; Holm Graessner, PhD; Norbert Kovacs, MD, D.Sc.; Anne Koy, MD, PhD; Belen Perez-Dueñas, MD, PhD; Carola Reinhard, PhD; Marina AJ Tijssen, MD, PhD; Sylvia Boesch, MD

*Contributed equally to this work.

Correspondence to:
Drs. L.M. Centen
Department of Neurology, University of Groningen, University Medical Center Groningen, Groningen, the Netherlands
Expertise Center Movement Disorders Groningen, University of Groningen, University Medical Centre Groningen, Groningen, the Netherlands
PO Box 30001, 9700 RB, Groningen
The Netherlands
Telephone: +31 50 361 61 61
E-mail: l.m.centen@umcg.nl

MANAGEMENT OF DYSTONIAS IN EUROPE

SURVEY

- **PART I.** PARTICIPANT´S CHARACTERIZATION

1. IDENTIFICATION

1.1. (surname, first name)

1.2. (e.g. neurologist, neurosurgeon, child-neurologist, …)

1.3. (e-mail)

1.4. Country (please tick the correspondent box below):

Austria	Belguim	Bulgaria	Croatia	Cyprus	Czech Republic	Denmark	Estonia
Finland	France	Germany	Greece	Hungary	Ireland	Italy	Latvia
Lithuania	Luxembourgh	Malta	Netherlands	Poland	Portugal	Romania	Slovakia
Slovenia	Spain	Sweden	UK	Norway			

1.5 Your main area of interest in dystonias is… (please tick only one box)

Genetics	Neurophysiology	Imaging	Clinical	Basic research	Botulinum toxin

Surgery	Clinical trials	Clinical scales	DBS	Rehabilitation	Other*

(*Please specify)

*________________________________

1.6 You are paediatrician/paediatric neurologist/neurologist/other (please tick only one box)

Paediatrician	Paediatric neurologist	Adult neurologist (seeing children)	Adult neurologist (not seeing children)	Other*

(*Please specify)

*__________________________

- **PART II.** COUNTRY CHARACTERIZATION

Note. The following questions should be answered by taking into account **your country** and not only your institution or city

2.1 How would you classify the accessibility of experts by patients with dystonias in **your country**? (please tick only one box)

Difficult	Satisfactory	Easy	Comments
			Why?

2.2 Regarding Movement Disorders (MDs) in general, is there in **your country**…

	No	Yes
An official MD society/ working group.............................		
MD experts..		
Stages in MD for residents (rotation, clinical fellowship) in Neurology...........................		
Teaching courses/symposia on MDs for residents/general neurologists..		
Teaching courses on MD for general practitioners..............		

Specific educational training on MD / dystonia available for:

Clinical nurse specialists..		
Speech therapists..		
Physiotherapists		
Clinical trials on dystonia		

2.3 Regarding Movement Disorders (MD) in **children**, is there in **your country**…

	**No**	**Yes**
An official MD working group for kids............................		
Child neurologists, MD experts for children......................		
Stages (rotation, clinical fellowship) on MD for residents in Pediatrics....................		
Teaching courses/symposia on MD for residents/general neurologists...		
Teaching courses in MD for general practitioners..............		

2.3 Specifically for **DBS in Dystonia**, are there teams in **your country**…

	**No**	**Yes**
**Experts in these fields:**		
Dystonia/DBS/neurologists..		
Dystonia/DBS/neurosurgeons...		
Dystonia/DBS/child neurologists.......................		

Other constellations..................

Comments:

Specific teaching courses/symposiums on dystonia for residents/general neurologists

Dystonia ....

DBS and dystonia...

Paediadric MD and dystonia......................

Teaching courses for general practitioners/paediatricians

Dystonia only..

DBS (who? targets?)..

Comments:

Specific educational training are available for:

Clinical nurse specialists on

Dystonia ....

DBS ....

Physiotherapists on

Dystonia ....

DBS...

Speech therapists on

Dystonia ....

DBS...

A network group exists (e.g. lists of experts in dystonia, lists of botulinum toxin centres, lists of centers for DBS…) for

Dystonia ....

DBS...

Dystonia and DBS..		

Please specify:

A national patients association about

Dystonia...		
MD incl. Dystonias...		

Tertiary centres for management

Dystonia		
DBS for dystonias..		

Clinical trials in these conditions

Dystonia		
DBS for dystonias...		

National diagnostic and therapeutic guidelines available in your country for:

Dystonia		
DBS for dystonias..		
other		

Comments:

2.4 Are all patients with dystonia (with/without DBS) managed in tertiary **neurological** centres at least once in the course of the disease in **<u>your country</u>**? (please tick only one box)

**No**	
**Yes**	

2.5 Is there any type of research on Dystonia (with/without DBS) ongoing in **<u>your country</u>**?

	Basic	Clinical	Genetics	DBS in dystonia	Neuro-physiology	Imaging	Other *
**No**							
**Yes**							

Comments: * (Please specify)

____

2.6 How long does it usually take for your patients from the **first appearance of dystonic symptoms until the first evaluation by a Movement Disorders** expert?

Less than 1 year
1-2 years
3-4 years
4 years or more

2.7 How long does it usually take from the **first evaluation to establishing a clinical diagnosis**?

Less than 1 year
1-2 years
3-4 years
>4-10 years
> 10 years

2.8. How long does it usually take from **first evaluation to offering a molecular diagnosis**?

Less than 1 year

1-2 years
3-4 years
4 years or more
I don’t seek for molecular confirmation

2.9 What percentage of your patients with Dystonia has a **genetic confirmation**?

>80%
>50 – 80%
30 – 50%
<30%
I don’t seek for molecular confirmation

3.0 What ancillary tests for Dystonia are available in **your country**?

**Ancillary tests**	**Easily accessible**	**Accessible with some difficulty**	**Not available**	**Comments**
Genetics (e.g. TOR1A, DYT6, PANK2, DYT 28 other: please specify)				
MRI				
Neurophysiological tests				
Others (Please specify)				

3.1 Which treatments are available/ accessible for dystonia in **your country?**

**Treatment**	**Easily accessible**	**Accessible with some difficulty**	**Not available**	**I don't know/ I'm not sure**	**Comments**
Medication (e.g. Anticholinergics, Baclofen,....)					
Botulinum toxin injection					
Deep brain stimulation					
Stereotactic lesioning (e.g. pallidotomy)					
Physical therapy and rehabilitation					
Speech therapy					
Occupational therapy					
Psychologist					
Human geneticist / genetic counselor					
Psychiatrist					
Social care worker					
Other (please specify)					

3.2 What percentage of your patients with **dystonia underwent DBS** in your country?

>20
10 – 20%
5% - 10%
<5%
None

3.3 What is the mean average age of your patients at time of DBS surgery for dystonia

6-10 years

10-15 years

15-20 years

>20 years

3.4 What is the most frequent cause of dystonia of your patients who underwent DBS

Primary (isolated) dystonia (with/without genetic confirmation)

Combined dystonia (with another movement disorder)

Complex dystonias (with other neurological/systemic manifestations)

Acquired dystonia (cerebral palsy, kernicterus, birth anoxia, infections…)

3.5 What kind of technical devices are available for patients with dystonia in your country, which are funded by the health system?

**Technical aids and devices**	**Easily accessible**	**Accessible with some difficulty**	**Not available**	**I don't know/ I'm not sure**	**Comments**
Manual wheelchair					
Electric wheelchair					
Assistive technology for communication (e.g talking mats…)					
Other (please specify)					

PART. III - PARTICIPANT´S OPINION

In your opinion, what are the 3 main issues/measures that should be urgently implemented for a better management of dystonia patients in **your country**?

1. Availability of genetic testing (early treatment options if available)

2. Multidisciplinary programms to enhance awareness of dystonia and treatment options in dystonias (adults/children)

3. Registries on patients (national, international)

____

____

____

____

____

Many thanks for your collaboration.
