# Supplementary material for: Dystonia management across Europe within ERN-RND: current state and future challenges
Source: J Neurol. 2022 Oct 6;270(2):797–809. doi: 10.1007/s00415-022-11412-4 (PMC9540051; doi:10.1007/s00415-022-11412-4)
Supplement: Supplementary file 2 — Supplementary file2 (DOCX 22 KB) [file 415_2022_11412_MOESM2_ESM.docx]

**Dystonia management across Europe within ERN-RND: current state and future challenges**

Journal of Neurology

Liesanne M. Centen^a,b^, MD*; David Pinter^c^, MD, PhD*; Martje E. van Egmond^a,b^, MD, PhD; Holm Graessner^d^, PhD; Norbert Kovacs^c^, MD, D.Sc.; Anne Koy^e^, MD, PhD; Belen Perez-Dueñas^f^, MD, PhD; Carola Reinhard^d^, PhD; Marina AJ Tijssen^a,b^, MD, PhD; Sylvia Boesch^g^, MD

*Contributed equally to this work.

**Correspondence to:**

Drs. L.M. Centen

^1^Department of Neurology, University of Groningen, University Medical Center Groningen, Groningen, the Netherlands

^2^Expertise Center Movement Disorders Groningen, University of Groningen,

University Medical Centre Groningen, Groningen, the Netherlands

PO Box 30001, 9700 RB, Groningen

The Netherlands

Telephone: +31 50 361 61 61

E-mail: l.m.centen@umcg.nl

| **Country** | **Participating center** |
| --- | --- |
| Austria | Center for Rare Movement Disorders / Dpt. of Neurology, Medical University Innsbruck, Innsbruck |
|  | Center for Pediatric Rare Neurological Diseases / Dpt. of Pediatrics, Medical University of Vienna, Vienna |
| Belgium | Antwerp University Hospital, Antwerp |
|  | University Hospitals Leuven, Leuven |
| Bulgaria | University Neurological Hospital “St. Naum”, Sofia |
| Croatia | University Hospital Center Zagreb Department of Neurology, Zagreb |
| Cyprus | The Cyprus Foundation for Muscular Dystrophy Research (The Cyprus Institute of  Neurology and Genetics) |
| Czech Republic | Motol University Hospital, Prague |
|  | Thomayer Hospital, Prague |
| Denmark | Aarhus Universitetshospital, Aarhus |
|  | Rigshospitalet, Copenhagen |
| Estonia | Tartu University Hospital, Tartu |
| Finland | Oulu University Hospital, Oulu |
| France | Assistance Publique-Hôpitaux de Paris, Hôpital Pitié-Salpêtrière, Paris |
| Germany | Universitätsklinikum, Aachen |
|  | Hannover Medical School, Hannover |
|  | Universitätsklinikum Schleswig-Holstein |
|  | Klinikum der Universität München, München |
|  | Universitätsklinikum Tübingen, Tübingen |
|  | Universitätsklinikum Würzburg, Würzburg |
| Greece | Eginitio Hospital, National and Kapodistrian, University of Athens, Athens |
| Hungary | University of Pécs, Pécs |
|  | Szent-Györgyi Albert Medical Center, University of Szeged, Szeged |
| Ireland | Tallaght University Hospital, Dublin |
| Italy | Azienda USL di Bologna - IRCCS Istituto delle Scienze Neurologiche, Bologna |
|  | Azienda Ospedaliera di Padova, Padova |
|  | Pediatric hospital Bambino Gesù, Rome |
|  | Azienda Ospedaliera Univesitaria Siena, Siena |
| Latvia | Pauls Stradins Clinical University Hospital, Riga |
| Luxembourg | Centre Hospitalier du Luxembourg, Luxenbourg |
| Malta | Mater Dei Hospital, Malta |
| The Netherlands | University Medical Center Groningen, Groningen |
|  | Maastricht University Medical Center, Maastricht |
| Poland | Krakow University Hospital, Krakow |
| Slovenia | University Medical Centre Ljubljana, Ljubljana |
| Spain | Hospital Clínic i Provincial de Barcelona y Hospital de Sant Joan de Déu, Barcelona |
|  | Hospital General Universitario Gregorio Marañón, Madrid |
|  | Hospital Universitario La Paz, Madrid |
|  | Hospital Clinico San Carlos, Madrid |
|  | Hospital Universitario Central de Asturias, Asturias |
|  | Hospital Universitario Marqués de Valdecilla, Cantabria |
|  | Hospital Universitari Vall d'Hebron, Barcelona |
| Sweden | Sahlgrenska Universitetssjukhuset, Göteborg |
| United Kingdom | University College London Hospitals NHS Foundation Trust, London |
