# Supplementary material for: Dystonia management across Europe within ERN-RND: current state and future challenges
Source: J Neurol. 2022 Oct 6;270(2):797–809. doi: 10.1007/s00415-022-11412-4 (PMC9540051; doi:10.1007/s00415-022-11412-4)
Supplement: Supplementary file 3 — Supplementary file3 (DOCX 23 KB) [file 415_2022_11412_MOESM3_ESM.docx]

**Dystonia management across Europe within ERN-RND: current state and future challenges**

Journal of Neurology

Liesanne M. Centen^a,b^, MD*; David Pinter^c^, MD, PhD*; Martje E. van Egmond^a,b^, MD, PhD; Holm Graessner^d^, PhD; Norbert Kovacs^c^, MD, D.Sc.; Anne Koy^e^, MD, PhD; Belen Perez-Dueñas^f^, MD, PhD; Carola Reinhard^d^, PhD; Marina AJ Tijssen^a,b^, MD, PhD; Sylvia Boesch^g^, MD

*Contributed equally to this work.

**Correspondence to:**

Drs. L.M. Centen

^1^Department of Neurology, University of Groningen, University Medical Center Groningen, Groningen, the Netherlands

^2^Expertise Center Movement Disorders Groningen, University of Groningen,

University Medical Centre Groningen, Groningen, the Netherlands

PO Box 30001, 9700 RB, Groningen

The Netherlands

Telephone: +31 50 361 61 61

E-mail: l.m.centen@umcg.nl

***Table S1*** *Availability of several types of allied healthcare providers or therapies per country: ‘+’ = easy availability. ‘+*’ = in some regions easily available, but not all regions of the country, ‘+-’ = with some difficulty available, ‘-’ = not available*

| **Country** | **Types of allied healthcare professionals or therapies** | | | | | | |
| --- | --- | --- | --- | --- | --- | --- | --- |
|  | **Physical therapy and rehabilitation** | **Speech therapy** | **Occupational therapy** | **Social care worker** | **Psychologist** | **Human geneticist/ counselor** | **Psychiatrist** |
| **Austria** | +* | +* | +* | + | +* | +* | +* |
| **Belgium** | +* | + | +- | +* | +* | + | + |
| **Bulgaria** | + | +- | +- | +- | +- |  | +- |
| **Croatia** | + | +- | +- | + | +- | +- | + |
| **Cyprus** | +- | +- | - | + | +- | +- | +- |
| **Czech Republic** | +* | +- | +- | +- | +* | +* | +* |
| **Denmark** | + | + | +* | + | +* | +* | + |
| **Estonia** | + | + | +- | + | +- | + | + |
| **Finland** | + | + | + | + | + | + | + |
| **France** | +* | +- | +* | +* | +* | +* | +- |
| **Germany** | +* | +* | +* | +* | +* | +* | +* |
| **Greece** | +- | +- | +- | +- | +- | +- | + |
| **Hungary** | +* | +* | +* | + | +* | + | + |
| **Ireland** | + | + | + | +* | +* | +- | +- |
| **Italy** | +- | +- | +- | +- | +* | +* | +* |
| **Latvia** | +- | +- | +- | +- | + | +- | + |
| **Luxembourg** | + | + | + | + | + | + | + |
| **Malta** | + | + | + | +- | +- | +- | +- |
| **Netherlands** | +* | +* | +* | +* | +* | + | +* |
| **Poland** | +- | +- | +- | +- | +- | +- | +- |
| **Slovenia** | + | + | + | +- | + | + | +- |
| **Spain** | +* | +* | +* | +* | +* | +* | +- |
| **Sweden** | + | + | + | + | + | + | + |
| **United Kingdom** | + | + | + | + | + | +- | + |
